# Development of an RGB-depth camera-based gait analysis system: a single-case study of a patient with stroke

**DOI:** 10.12701/jyms.2026.43.15

**Published:** 2026-01-24

**Authors:** Min Cheol Chang, Juyeon Kim, Jun Sung Moon, Wooktae Park, Gun Woo Lee, Yoo Jin Choo

**Affiliations:** 1 Department of Physical Medicine and Rehabilitation, Yeungnam University College of Medicine, Daegu, Korea; 2 A-ZoneTech, Daegu, Korea; 3 Division of Endocrinology and Metabolism, Department of Internal Medicine, Yeungnam University College of Medicine, Daegu, Korea; 4 Department of Orthopaedic Surgery, Yeungnam University College of Medicine, Daegu, Korea

**Keywords:** Camera, Gait, Gait analysis, Remote sensing technology, Stroke

## Abstract

Alterations in gait patterns often indicate health status, and their analysis enables the diagnosis and assessment of various health conditions. This study aimed to develop a noncontact gait analysis system using red, green, and blue-depth (RGB-D) cameras and to evaluate its potential clinical applicability. A single case study was conducted to assess changes in the gait patterns of a patient with stroke before and after the application of an ankle-foot orthosis. Twenty walking trials were recorded to evaluate the key gait parameters. The custom RGB-D camera-based gait analysis system demonstrated the potential to rapidly quantify key gait parameters in the patient. Compared with normative data, it effectively identified characteristic stroke-related gait impairments such as shorter step lengths and slower gait speeds. However, the intraclass correlation coefficient analysis indicated low measurement reliability. Although the stance time and minimum knee angle on the left and right sides exceeded the standard error of measurement (SEM), no changes exceeded the minimal detectable change (MDC) criteria. Moreover, other gait parameters did not show significant changes beyond SEM or MDC, limiting the interpretability of the results. Therefore, further technological developments and data collection are required to improve test-retest reliability and sensitivity to change.

## Introduction

Gait is one of the most fundamental human movements and is essential for maintaining independence and quality of life in daily activities. Normal gait arises from the complex interplay between multiple systems, including musculoskeletal, nervous, and sensory systems. Consequently, abnormalities in these systems can lead to alterations in gait patterns and serve as key indicators of an individual’s overall health status.

Stroke is a major central nervous system disorder, with gait impairment affecting >50% of patients [1]. Gait disorders in patients with stroke primarily result from neurological impairments such as muscle weakness, paralysis, balance deficits, and spasticity [2]. Spasticity is a common post-stroke complication that can disrupt normal gait by limiting the natural movements of the ankle, knee, and hip joints [3]. Post-stroke gait impairment reduces walking independence, increases the risk of falls, and adversely affects quality of life [1]. Therefore, accurate assessments and management using both qualitative and quantitative methods are essential.

Qualitative assessment tools based on clinical observations include the Functional Ambulation Category (FAC) for evaluating ambulatory independence, the Nottingham Sensory Assessment (NSA) for sensory function, and the Modified Ashworth Scale (MAS) for muscle tone. Although widely used in clinical practice, these assessments are subject to evaluator bias, with outcomes varying depending on the assessor’s experience and proficiency. Furthermore, these tools are limited in detecting subtle functional changes and identifying detailed gait characteristics. Therefore, quantitative assessment methods have been introduced to overcome these limitations. Among these methods, the marker-based three-dimensional (3D) motion analysis system is the most widely used method for quantitative gait analysis. Although it is highly precise in measuring gait patterns, it involves attaching markers to the patient, resulting in long preparation times and potential discomfort. Additionally, its reliance on specialized equipment and laboratory settings leads to high installation and maintenance costs, requiring skilled personnel for operation and data analysis. These limitations substantially hinder its accessibility in clinical settings. Other methods, including wearable sensor-based systems and electromyography-based gait analyses, have also been used [4,5]. However, they present challenges, such as the time-consuming attachment of electromyography electrodes and discomfort during prolonged use. Moreover, factors such as sweating and muscle fatigue can affect data accuracy during walking [4,6].

To address the limitations of conventional gait analysis methods, there is an increasing interest in the development of more convenient and efficient techniques that do not require attaching sensors or markers to the patient. One such method utilizes red, green, and blue-depth (RGB-D) cameras for noncontact gait analysis. Previous studies have shown that using a red, green, and blue (RGB) camera alone limits gait analysis from the frontal plane because of the lack of depth information, necessitating sagittal plane measurements [7]. Similarly, depth cameras alone are prone to data loss or noise from infrared interference, which can reduce the interpolation accuracy [8]. An RGB-D camera overcomes these limitations by integrating color, shape, and 3D data, thereby enabling more stable joint tracking and motion analysis [9]. A noncontact gait analysis method using an RGB-D camera minimizes patient discomfort and captures natural gait patterns without the need for physical attachments. RGB-D cameras provide both RGB images and depth information, enabling the precise extraction of human movement in 3D coordinates. This capability facilitates effective analysis of key gait parameters, including gait speed, step length, gait symmetry, and joint angles. In addition, the ability to process and visualize data in real time enables immediate feedback in clinical settings. Unlike conventional systems, RGB-D cameras can automatically track the movements of various body parts without requiring dedicated markers or wearable devices, offering substantial installation advantages [10]. Thus, noncontact gait analysis using RGB-D cameras is a promising alternative to conventional methods because it provides a more convenient and comfortable solution for assessing gait patterns in clinical settings.

This study aimed to develop a novel noncontact gait analysis system that combines an RGB-D camera with custom analysis software and to explore its clinical applicability. Gait parameters were analyzed before and after wearing an ankle-foot orthosis (AFO) in a single patient with stroke.

## Case

**Ethics statement:** This study was exempted from review by the Institutional Review Board (IRB) of Yeungnam University Hospital (IRB No: YUMC 2024-09-021). Written informed consent was obtained from the patient to participate in the study.

### 1. Participant characteristics

The participant was a woman in her late 40s who was diagnosed with a left thalamic hemorrhage 14 years ago. She visited the Stroke Center of Yeungnam University Hospital in Daegu, Korea. The patient had right hemiplegia and exhibited a circumduction gait. Muscle strength was assessed using the Medical Research Council scale, which ranges from 0 (no muscle contraction) to 5 (normal strength). Her shoulder flexor, elbow flexor, wrist extensor, hip flexor, and knee extensor muscles were graded 3; finger flexor, 2; and ankle dorsiflexor, 1. The patient was classified as FAC 4 (walking independently on flat surfaces but requiring supervision on stairs and uneven surfaces). On the right side, the NSA subscale scores were 16/20 for tactile sensation and 20/24 for kinesthetic sensation. Right ankle plantar flexor spasticity was rated as 2 on the MAS, and a right ankle clonus of 2 beats to 3 beats was observed. The patient had no neuropathic pain and was not receiving medication, physical therapy, or occupational therapy. The patient had been using a right solid AFO since 2010 (Supplementary Fig. 1A). Gait analysis was performed to compare walking abilities with and without the AFO.

### 2. Gait analysis equipment and system configuration

**Fig. 1.**
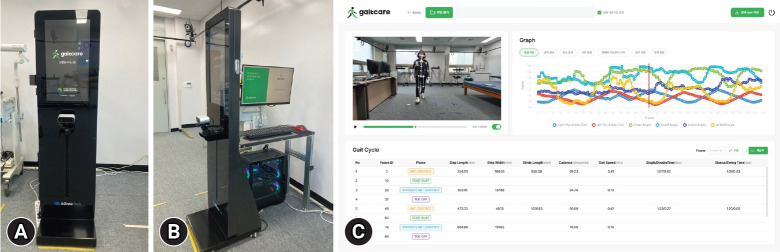
Gait analysis equipment. (A) Depth camera mounted 91 cm above the ground, displaying gait analysis instructions on the kiosk screen. (B) Real-time gait analysis system connected to a computer for immediate data processing. (C) Display of gait metrics and analysis results in real-time.

The gait analysis system used in this study consisted of an RGB-D camera (Femto bolt; Orbbec, Troy, MI, USA) and a computer for processing the camera data. The RGB-D camera operated in a narrow field-of-view mode with a resolution of 640×576 at 30 frames per second, whereas the RGB camera captured images at a resolution of 3,840×2,160 using the Motion JPEG format at 30 frames per second. The computer was equipped with an i7-14700K (Intel, Santa Clara, CA, USA) central processing unit, 64 GB of random access memory, and an RTX 4090 graphics processing unit (NVIDIA, Santa Clara, CA, USA). For the gait analysis, the camera was positioned to capture the frontal view of the participant’s gait path. The equipment setup allowed the analyst to control the analysis software from the side, where the start and end points of the gait path could be clearly observed (Fig. 1A, 1B). The software used for the analysis was developed using algorithms written in C++ and a user interface/user experience designed in C# (Fig. 1C). The software was installed on a computer under the name “GaitCare.”

### 3. System workflow and measurement procedure

**Fig. 2.**
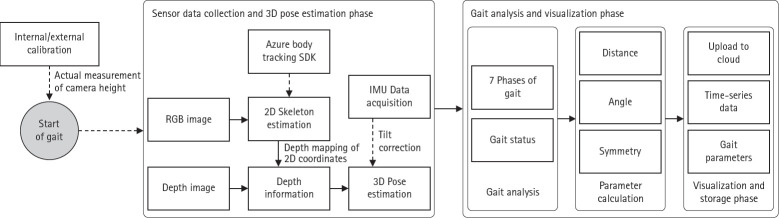
Flowchart illustrating the noncontact gait analysis system. 3D, three-dimensional; SDK, Software Development Kit; 2D, two-dimensional.

The gait analysis system developed in this study collects real-time gait data using an RGB-D camera and the Azure Body Tracking Software Development Kit (Microsoft, Redmond, WA, USA) and computes biomechanical parameters through 3D gait analysis. The system comprises 5 main stages: (1) system initialization and calibration, (2) data acquisition, (3) 3D pose estimation, (4) gait analysis and parameter computation, and (5) visualization and cloud-based storage. By integrating hardware and software, this system enables repetitive and quantitative analyses. A flowchart of the system is shown in Fig. 2.

### 4. System initialization and camera calibration

Before use, the RGB-D camera was calibrated internally and externally to ensure its accuracy. Internal calibration aligned the RGB camera and depth sensor, including the focal length, optical center, and distortion coefficients. External calibration adjusted the position and orientation of the device relative to a fixed-coordinate system. Additionally, the system utilized the built-in inertial measurement unit (IMU) of the RGB-D camera to detect the tilt and rotation of the device in real time, ensuring the vertical alignment of the depth sensor. After calibration, the RGB, depth, and IMU sensors were synchronized to produce an integrated dataset at the frame level. The graphical user interface (GUI) displayed the device connection status and calibration results. The user then instructed the individual to walk naturally. During the trial, the RGB-D camera captured RGB videos, depth maps, and IMU signals and synchronized them into cohesive frames for subsequent gait analysis. The data were streamed in real time to the software, enabling continuous tracking of postural changes throughout the gait cycle.

### 5. Skeleton estimation and three-dimensional pose calculation

Skeleton estimation was performed by first predicting the two-dimensional (2D) joint coordinates from the RGB video, which were then mapped onto a depth map to compute the final 3D pose. Using the Azure Body Tracking Software Development Kit, the system identified key joint locations (e.g., hip, knee, and ankle) in the 2D frame and associated them with depth values to determine accurate 3D joint positions. The IMU-derived posture data were used to correct the coordinate system, ensuring stable skeleton estimation even in tilted environments. The resulting 3D pose data were processed in real time, frame-by-frame, for gait analysis.

### 6. Gait analysis and parameter calculation

Using 3D joint coordinates, various gait analysis algorithms were applied. The GaitCare system analyzed real-time data such as joint height (Y-axis), distance, and velocity. The step length was calculated from the repeated foot contact positions, and the stride length was determined from the distance between the left and right foot contacts. Walking speed was computed from the total distance and time, whereas cadence was derived from the gait cycle frequency over time. Additionally, single and double support durations were evaluated based on foot contact with the ground. The stance and swing phases were calculated by averaging the duration for which both feet were on and off the ground, respectively. The joint angles of the hip, knee, and ankle were calculated as shown in Supplementary Fig. 2. The hip angle was defined as the angle between the line connecting the neck (near the 7th cervical vertebra) and the hip, and the line between the hip and knee. The knee angle was defined as the angle between the lines connecting the hip to the knee and the knee to the ankle. The ankle angle was defined as the angle between the lines connecting the knee to the ankle and the ankle to the midpoint of the metatarsals. In this system, no specific criteria for the minimum or maximum joint angles were set, and the values generated according to the participant’s gait were recorded.

### 7. Visualization and cloud-based storage

Gait analyses were visualized in real time using a GUI. After completion of the measurement, the system provided outputs such as time and distance variation graphs for each gait phase, 3D gait trajectory sequences based on skeleton data, synchronized 2D video frames overlaid with corresponding 3D joint positions, and a summary report of the entire gait cycle.

All data were automatically saved in JavaScript Object Notation or comma-separated values format and uploaded to a cloud server, organized by screening number and measurement date. The cloud system enables long-term storage of accumulated patient data and trend analysis of gait changes. This allows medical professionals to quantitatively assess patient conditions over time.

### 8. Data collection and experimental procedure

The participant stood 4 m away from the camera installed at a height of 91 cm on a flat surface. The participant then walked in a straight line facing the camera. Recordings were made while the participant walked toward the camera; the return walk, with the participant facing away from the camera, was not recorded. A total of 20 trials were conducted, 10 with and 10 without the AFO, and the total time required for all recordings was 5 minutes. The participant walked without any assistive devices such as crutches or a cane. Data were collected using the software developed in our laboratory. After pressing the start button, a video of the participant walking was captured in real time and automatically input into the software, which extracted the skeleton data. When the participant walked 4 m toward the camera, one recording session was completed, and the corresponding data were saved to cloud storage. Subsequently, the captured video was analyzed using the software’s tools. The assessed gait parameters included the average step length, step width, stride length, cadence, gait speed, single support time, double support time, stance time, swing time, and the maximum and minimum angles for the hip, knee, and ankle joints on both sides based on each gait cycle. After data collection, the participant provided feedback on her experience using the noncontact gait analysis system.

The intraclass correlation coefficient (ICC), standard error of measurement (SEM), and minimal detectable change (MDC) were calculated to assess the measurement error and determine whether the changes in gait parameters before and after AFO application exceeded random variation. The ICC was calculated using a two-way random effects model with absolute agreement using R software version 4.2.1 (R Foundation for Statistical Computing, Vienna, Austria). In this study, the ICC primarily served as a reference for calculating the SEM rather than directly evaluating measurement reliability. This is because the study involved a single participant and assessor (GaitCare), which limited the interpretative value of the ICC in assessing reliability. Based on the calculated ICC, SEM was derived using the following formula:


$$SEM=\frac{SD_{\text{with }AFO}+SD_{\text{without }AFO}}{2}\times \sqrt{1-ICC}$$

Subsequently, the MDC at a 95% confidence level was calculated as:


$$MDC=SEM\times 1.96\times \sqrt{2}$$

A difference between the pre- and post-AFO measurements exceeding the calculated MDC was interpreted as a true change beyond the measurement error.

### 9. Observed gait outcomes

Analysis of gait parameters before and after AFO use revealed that the stance time and the minimum knee angles on both sides decreased, and the maximum ankle angle on the right side increased, all beyond the SEM. However, none of these changes exceeded the MDC. The variables that showed no significant differences based on SEM and MDC included step length, step width, stride length, cadence, gait speed, single and double support times, swing time, maximum and minimum hip joint angles on both sides, maximum knee joint angles on both sides, maximum and minimum angles of the left ankle joint, and minimum angle of the right ankle joint. These results suggest that although there was a trend toward changes in stance time, minimum angles of both knee joints, and maximum angle of the right ankle joint depending on AFO use, these changes were not clinically meaningful. Detailed gait analysis results before and after wearing the AFO are presented in Table 1. Participant feedback on the noncontact gait analysis system was highly positive, noting its convenience because there was no need for clothing changes or marker attachments and the assessment was completed quickly.

## Discussion

This study was conducted to evaluate the technical feasibility and practicality of an RGB-D camera-based gait analysis system using software developed in our laboratory. Unlike conventional marker-based gait analysis, the RGB-D camera-based system utilizes a noncontact approach that minimizes patient discomfort and enables the assessment of natural gait patterns [11]. Conventional gait analysis often involves attaching electrodes or markers to the patient’s body, a process that requires considerable preparation time and can cause discomfort [12]. Additionally, the use of electrodes may lead to skin issues [13]. Specialized personnel are required to conduct gait analysis, and the entire process, from preparation to analysis, typically requires approximately 1 hour, which poses challenges in busy clinical settings [11,12]. In contrast, the RGB-D camera-based gait analysis system eliminates the need for preparation, allowing the entire process to be completed in less than 5 minutes, thereby enhancing convenience. Real-time results can be displayed using software, enabling rapid assessment of the patient’s condition. Furthermore, conventional systems used in clinical settings often require more than six cameras, whereas the RGB-D camera-based system operates effectively with a single camera, offering considerable advantages in terms of installation. Considering these advantages, the RGB-D camera-based gait analysis system demonstrates substantial potential for clinical applications and may serve as a promising alternative to conventional contact-based gait analysis methods.

This study examined how gait parameters in a patient with stroke approached normative data from healthy adults, despite the challenge of achieving a completely normal gait with only an AFO. Such comparisons provide valuable insights into gait improvement and help verify the performance of the GaitCare system. Some results were compared with the normative data from the RGB-D camera-based gait analysis by Röhling et al. [14]. After wearing the AFO, the step length (433.68±88.45 mm) and step width (87.40±14.45 mm) were shorter than the normative values (693.5±76.9 and 100.19±27.2 mm, respectively). The cadence (113.86±49.13 steps/minute) was slightly lower than the normative value of 122.07±10.55 steps/minute. Gait speed (0.72±0.10 m/second) was slower than the normative value of 1.6±0.17 m/second, indicating a slower gait speed in the patient compared to that of healthy individuals. Although the gait parameters differed from the normative data, the participant demonstrated typical stroke-related gait characteristics such as shorter step length and slower gait speed. These findings suggest that the GaitCare system is a valid tool for identifying major gait abnormalities in patients with stroke. 

The noncontact gait analysis system developed in this study has limitations in terms of providing accurate results. This was evident, as no gait parameters showed changes exceeding the MDC, and repeated measurements lacked consistency. These limitations may be due to technical or environmental factors. Although AFOs are typically expected to reduce the plantar flexion angle to prevent foot drop, the data in this study showed an increase in the plantar flexion angle after AFO use. Additionally, the joint angles on the left (unaffected) side were generally smaller than those on the right (affected) side, contrary to the typical expectations in patients with hemiparesis. These unexpected findings may be attributed to the compensatory movement strategies commonly observed in patients with chronic stroke, in which the unaffected limb adopts a more guarded pattern to maintain stability. However, technical errors such as inaccurate joint point recognition or reduced detection accuracy during asymmetric gait patterns cannot be excluded. Environmental factors including lighting, clothing, and measurement distance may have also influenced the analysis. Overly bright ambient lighting may distort or weaken the depth signals because depth cameras rely on infrared light for distance measurements [15]. RGB cameras are highly sensitive to lighting; thus, in dark environments, noise can degrade the image quality, making posture recognition difficult [16]. Moreover, loose pants or skirts could hinder joint data collection [17]. Furthermore, if the participant is too close or too far from the camera, either the full body may not be captured or the distance signals may weaken, resulting in data errors. Considering the sensitivity of RGB-D cameras to environmental factors, these vulnerabilities should be considered in clinical applications.

A major limitation of this study is its single-participant design, which was used to identify technical errors and examine the stability of the RGB-D camera-based noncontact gait analysis system during the initial validation phase. Unlike conventional marker-based methods, this system operates in a noncontact manner using RGB-D imaging. Therefore, before conducting a large-scale study, it is essential to validate the basic operating principles and analytical processes of the system. A patient with stroke and observable hemiparetic gait characteristics was selected for the study, and repeated data collection and analysis were performed to identify the functional limitations of the GaitCare system for improvement. Future studies will include a larger number of patients with stroke and healthy individuals to expand the gait data and enhance system performance through algorithm advancements.

Moreover, we are expanding our research to include healthy participants and patients with neurological or musculoskeletal conditions such as cervical myelopathy and scoliosis to refine the analysis algorithms. Technical updates are also underway to enable more precise gait data analysis as follows: (1) in the phase-specific gait cycle analysis, the gait cycle will be subdivided into phases, including initial contact, mid-stance, terminal stance, pre-swing, mid-swing, and terminal swing, to provide more detailed and granular data; (2) for assessments of balance and movement stability, hip vertical displacement and pelvic rotation angles will be calculated relative to the second sacral vertebra; and (3) in circumduction gait detection, a calculation method will be introduced to identify circumduction by analyzing the hip abduction angle on left and right sides during a gait cycle. Advancements in RGB-D camera technology are expected to contribute to the refinement of gait analysis algorithms. Furthermore, RGB-D camera-based gait analysis systems are significantly influenced by the performance of the analysis systems and advancements in algorithms [18]. As the performance of analysis software and algorithms continues to improve, more accurate data processing and analysis are expected, leading to improved precision of gait analysis results. Currently, the algorithm for distinguishing the detailed phases of the gait cycle is based on extracted data values. Therefore, experts must verify whether the frames identified by the algorithm accurately represent the gait cycle phases. This process may require a large volume of data to be reviewed at 30 frames per second, potentially resulting in limitations related to time and personnel. However, these challenges are expected to be addressed through advancements in RGB-D camera technology, algorithm refinement, and the accumulation of sufficient sample data.

The proposed RGB-D camera-based cnoncontact gait analysis system is expected to serve as an effective evaluation tool for the rehabilitation of patients with stroke. This system could substantially enhance the accessibility and practicality of clinical gait analysis by enabling a more precise assessment of patient conditions and contributing to the development of optimized rehabilitation plans. Moreover, this system has potential applicability (beyond patients with stroke) to other conditions associated with an abnormal gait [10,19]. For instance, it could be valuable to compare pre- and post-surgical gait abilities and monitor recovery processes in musculoskeletal conditions, such as joint replacement surgeries or fracture treatments. It could also facilitate the quantitative analysis of muscle coordination and gait asymmetry in neurological disorders, including Parkinson’s disease, spinal cord injuries, and cerebral palsy. Additionally, the system may effectively evaluate gait instability associated with aging, thereby contributing to health management and preventive care for adults who are older. In summary, the RGB-D camera-based noncontact gait analysis system is expected to have broad clinical applicability, including gait monitoring, pre- and post-treatment evaluation, and personalized rehabilitation planning for various conditions. This system has the potential to become an innovative assessment tool that can address the limitations of conventional gait analysis methods.

## Figures and Tables

**Table 1.** Results of gait analysis

Variable	Without AFO	With AFO	ICC	SEM	MDC
Step length (mm)	446.31±46.18	433.68±88.45	0.372	53.34	147.84
Step width (mm)	97.22±19.98	87.40±14.45	0.507	13.64	37.81
Stride length (mm)	908.00±78.06	866.59±219.13	0.229	117.74	326.36
Cadence (steps/min)	92.60±20.88	113.86±49.13	0.353	27.73	76.87
Gait speed (m/sec)	0.66±0.08	0.72±0.10	0.185	0.07	0.19
Single support time (sec)	1.10±0.17	0.97±0.21	0.369	0.15	0.41
Double support time (sec)	0.33±0.06	0.30±0.08	–0.140	0.05	0.15
Stance time (sec)	0.95±0.16	0.78±0.21	0.411	0.15	0.41
Swing time (sec)	0.48±0.12	0.48±0.11	–0.428	0.09	0.26
Hip angle					
Left_max	44.43±5.81	48.01±4.61	–0.469	4.13	11.44
Left_min	0.25±0.25	0.24±0.23	0.046	0.19	0.52
Right_max	45.06±9.27	44.87±6.69	–0.397	6.32	17.52
Right_min	0.16±0.17	0.31±0.30	–0.369	0.19	0.52
Knee angle					
Left_max	175.34±2.34	175.59±2.03	–0.193	1.73	4.8
Left_min	105.24±7.71	98.30±7.36	–0.142	5.97	16.55
Right_max	177.61±0.76	177.66±0.86	0.227	0.64	1.77
Right_min	117.23±11.75	108.44±6.88	–0.274	7.38	20.46
Ankle angle					
Left_max	139.92±4.97	139.76±2.96	0.370	3.14	8.71
Left_min	79.95±8.44	78.86±5.06	–0.156	5.35	14.82
Right_max	149.45±7.58	159.55±5.91	0.061	5.34	14.81
Right_min	50.30±4.31	48.01±3.32	0.034	3.03	8.39

AFO, ankle-foot orthosis; ICC, intraclass correlation coefficient; SEM, standard error of measurement; MDC, minimal detectable change; max, maximum; min, minimum.

## Supplementary materials

Supplementary Figs. 1 and 2 can be found at https://doi.org/10.12701/jyms.2026.43.15.

Supplementary Fig. 1. Ankle-foot orthosis worn by a patient with stroke, and gait patterns of a patient with stroke. (A) Ankle-foot orthosis. (B) Walking without an ankle-foot orthosis. (C) Walking with an ankle-foot orthosis.

Supplementary Fig. 2. Criteria for calculating the hip, knee, and ankle joint angles
