# Supplementary material for: Development of an RGB-depth camera-based gait analysis system: a single-case study of a patient with stroke
Source: J Yeungnam Med Sci. 2026 Jan 24:jyms.2026.43.15. doi: 10.12701/jyms.2026.43.15 (PMC12957859; doi:10.12701/jyms.2026.43.15)
Supplement: Supplementary Fig. 1. — Ankle-foot orthosis worn by a patient with stroke, and gait patterns of a patient with stroke. (A) Ankle-foot orthosis. (B) Walking without an ankle-foot orthosis. (C) Walking with an ankle-foot orthosis. [file jyms-2026-43-15-Supplementary-Fig-1.pdf]

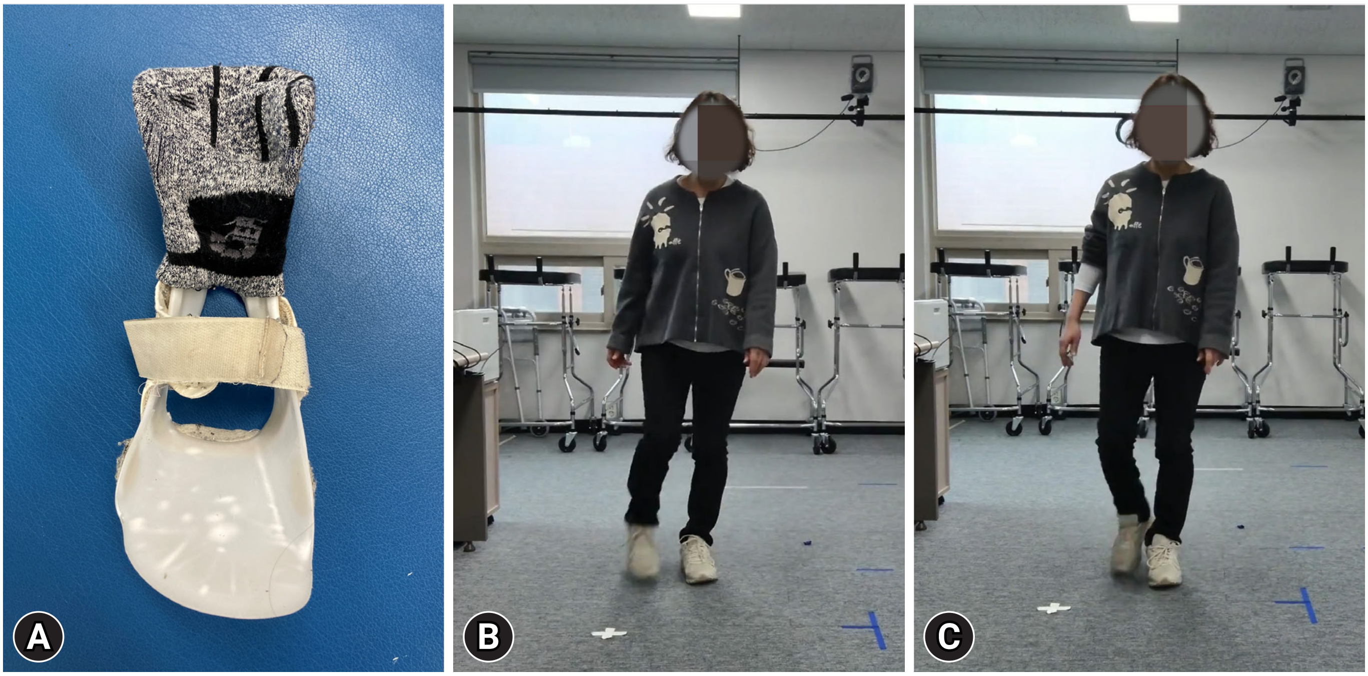

Supplementary Fig. 1. Ankle-foot orthosis worn by a patient with stroke, and gait patterns of a patient with stroke. (A) Ankle-foot orthosis. (B) Walking without an ankle-foot orthosis. (C) Walking with an ankle-foot orthosis.
