# Supplementary figures and images for: Development of an RGB-depth camera-based gait analysis system: a single-case study of a patient with stroke
Source: J Yeungnam Med Sci. 2026 Jan 24:jyms.2026.43.15. doi: 10.12701/jyms.2026.43.15 (PMC12957859; doi:10.12701/jyms.2026.43.15)

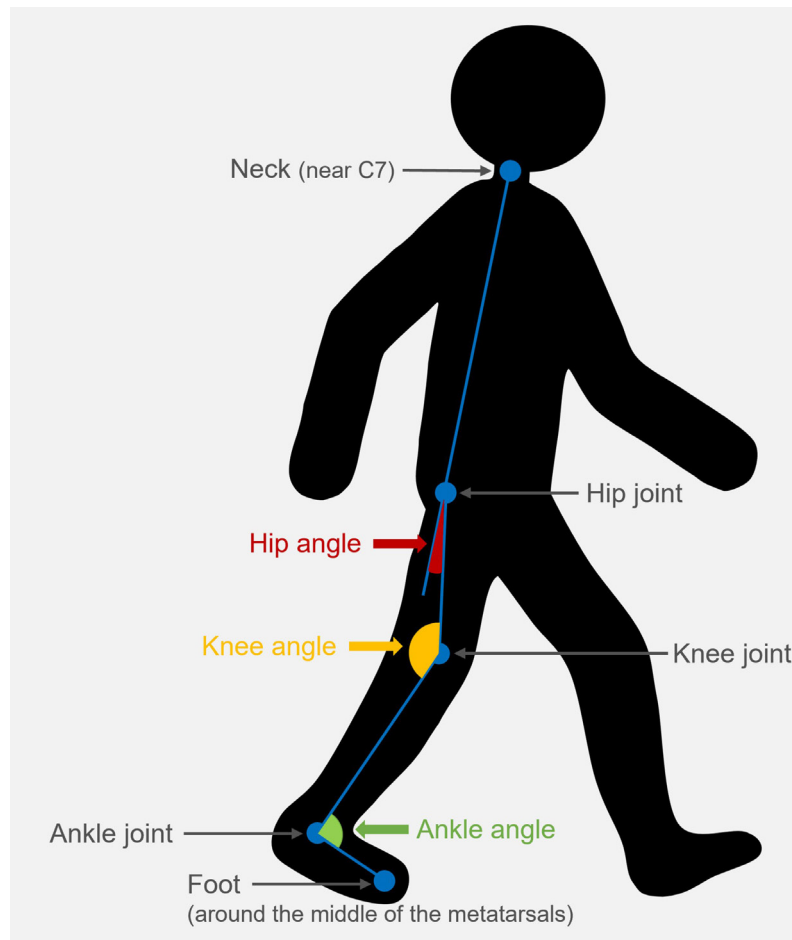

**Supplementary Fig. 2.** Criteria for calculating the hip, knee, and ankle joint angles.

Supplement: Supplementary Fig. 2. — Criteria for calculating the hip, knee, and ankle joint angles [file jyms-2026-43-15-Supplementary-Fig-2.pdf]
